# Low‐Dose Mavacamten Initiation in Obstructive Hypertrophic Cardiomyopathy: A Real‐World Study in China

**DOI:** 10.1155/cdr/5690104

**Published:** 2026-01-14

**Authors:** Wenlong Yang, Hui Shi, Rebecca Suchi Chang, Yin Zhang, A. Jixiang, Chunyu Wu, Juying Qian, Junbo Ge, Shuning Zhang

**Affiliations:** ^1^ Department of Cardiology, Zhongshan Hospital, and Shanghai Institute of Cardiovascular Diseases, Fudan University, Shanghai, China, fudan.edu.cn; ^2^ State Key Laboratory of Cardiovascular Diseases, Zhongshan Hospital, Fudan University, Shanghai, China, fudan.edu.cn; ^3^ NHC Key Laboratory of Ischemic Heart Diseases, Fudan University, Shanghai, China, fudan.edu.cn; ^4^ Key Laboratory of Viral Heart Diseases, Chinese Academy of Medical Sciences, Shanghai, China, cacms.ac.cn; ^5^ National Clinical Research Center for Interventional Medicine, Shanghai, China; ^6^ Department of Cardiology, Shanghai Geriatric Medical Center, Shanghai, China

**Keywords:** left ventricular outflow tract obstruction, mavacamten, obstructive hypertrophic cardiomyopathy

## Abstract

**Aims:**

To evaluate the real‐world efficacy and safety of low‐dose (2.5 mg) mavacamten initiation in Chinese patients with symptomatic obstructive hypertrophic cardiomyopathy (oHCM).

**Methods and Results:**

This single‐center observational study (Zhongshan Hospital, China; Oct 2024–Apr 2025) enrolled 72 symptomatic oHCM patients (NYHA II/III, LVEF ≥ 55%, resting/Valsalva‐provoked LVOT gradient [LVOTG] ≥ 30 mmHg). All patients initiated mavacamten 2.5 mg once daily. Doses were adjusted at Weeks 4, 8, and 12 based on LVEF and LVOTG. Primary outcomes were changes in resting/provoked LVOTG, NT‐proBNP, and NYHA class at Week 12. Safety outcomes included LVEF < 50%, cardiac hospitalization, and death.

Significant reductions from baseline to Week 12 were observed: Resting LVOTG (52.4 ± 28.7 to 32.1 ± 23.1 mmHg, *p* < 0.001), Valsalva‐provoked LVOTG (74.1 ± 24.4 to 48.7 ± 25.4 mmHg, *p* < 0.001), NT‐proBNP (1102.7 ± 1114.9 to 320.2 ± 406.2 pg/mL, *p* < 0.001). LVEF remained stable. NYHA class improved by ≥ 1 class in 83.3% (60/72) of patients.

Subgroup analyses revealed significantly greater LVOTG reductions in patients with classic HCM (vs. apical HCM, *p* < 0.001) and high baseline resting LVOTG (≥ 50 mmHg vs. < 50 mmHg, *p* < 0.001/*p* = 0.04). NYHA improvement was consistent across subgroups.

Twenty‐two patients escalated to 5 mg at Week 12, achieving further significant LVOTG reductions (*p* < 0.001), particularly in apical HCM and high‐baseline‐gradient subgroups, with stable LVEF.

No safety events occurred (LVEF < 50%, arrhythmias, hospitalization, and death). Four patients reported transient minor adverse events (dizziness, nausea, and fatigue).

**Conclusion:**

In this first Chinese real‐world study, initiating mavacamten at 2.5 mg significantly improved haemodynamics (LVOTG), biomarkers (NT‐proBNP), and functional status (NYHA) in oHCM patients over 12 weeks with an excellent safety profile. Greater haemodynamic efficacy was observed in classic HCM and high‐baseline‐gradient patients. Escalation to 5 mg provided additional benefit. Mavacamten is an effective and safe therapy for oHCM in this Asian population.

## 1. Introduction

Hypertrophic cardiomyopathy (HCM) is a genetic cardiac disorder characterized by left ventricular hypertrophy, occurring in the absence of other cardiovascular diseases or systemic and metabolic diseases known to cause ventricular wall thickening. The primary cause of HCM is genetic mutations in genes encoding sarcomeric proteins or sarcomere‐associated structural proteins, and it is mainly inherited in an autosomal dominant pattern [1]. It is estimated that the prevalence of HCM in the adult population ranges from 1:200 to 1:500 [2]. The distribution and severity of ventricular wall thickening exhibit considerable heterogeneity among HCM patients. Approximately 60%–70% of patients present with obstructive left ventricular outflow tract (LVOTO), which is defined as a left ventricular outflow tract gradient (LVOTG) ≥ 30 mmHg at rest or on provocation. LVOTO increases left ventricular systolic pressure and aggravates myocardial ischemia, which is the main cause of symptoms in patients with obstructive HCM (oHCM). LVOTO also significantly increases the risk of adverse events, including sudden cardiac death, heart failure progression, stroke, and death in HCM patients [3, 4].

For decades, oHCM management has focused on symptom alleviation and functional improvement [4]. Currently, first‐line pharmacological therapy, retaining a Class I guideline recommendation, comprises *β*‐blockers and nondihydropyridine calcium channel blockers. Disopyramide, which exerts negative inotropic and chronotropic effects, effectively reduces LVOTG and may improve diastolic function in oHCM. However, its utility is limited by anticholinergic side effects, reduction of cardiac function, significant QT interval prolongation, and poor tolerability in many patients. Additionally, disopyramide is unavailable in China and numerous Asian countries. While these medications modulate cardiac rhythm, provide negative inotropy, and improve symptoms and quality of life, they do not specifically target the underlying molecular pathogenesis or pathophysiology of HCM [5]. Consequently, they fail to halt disease progression or demonstrably reduce the incidence of sudden cardiac death. For patients with refractory symptomatic oHCM, invasive interventions such as surgical septal myectomy or alcohol septal ablation offer highly effective LVOTG reduction and symptom relief. However, their application is constrained by procedural invasiveness and dependence on operator expertise [6].

Mavacamten represents a novel therapeutic approach as a selective, reversible, allosteric inhibitor of cardiac myosin. It targets the ATPase activity of cardiac myosin heavy chain, stabilizing the myosin head in an “off” or “super‐relaxed” state, thereby preventing excessive myosin–actin cross‐bridge formation [7, 8]. Clinical trials, including EXPLORER‐HCM and VALOR‐HCM, have established that mavacamten significantly reduces LVOTG, improves exercise capacity, enhances New York Heart Association (NYHA) functional class, and ameliorates patient health status, demonstrating both safety and efficacy in oHCM [9, 10]. Notably, the Phase III EXPLORER‐CN trial conducted in China utilized a starting dose of 2.5 mg, distinct from the 5 mg starting dose used in other studies. This adjustment accounted for the lower average body weight and higher prevalence of CYP2C19 poor metabolizers within the Chinese population. Results confirmed mavacamten′s efficacy and safety profile in this cohort [11]. Based on this cumulative evidence, mavacamten received approval from the US Food and Drug Administration (FDA) in April 2022 and the National Medical Products Administration (NMPA) of China in April 2024 for the treatment of symptomatic oHCM in adults [12].

While mavacamten has been utilized globally for over 3 years, the majority of real‐world evidence originates from regions outside Asia, with a particular paucity of data from China. Chinese oHCM patients exhibit a higher incidence of CYP2C19 poor metabolizer status, a greater frequency of apical hypertrophy, and lower starting doses, factors potentially influencing mavacamten′s clinical application. Herein, we report our initial real‐world clinical experience with mavacamten in the management of oHCM.

## 2. Methods

### 2.1. Study Design and Population

This single‐center, observational study involved patients with HCM receiving treatment with mavacamten at Zhongshan Hospital, Fudan University in China. HCM was diagnosed according to current guidelines, defined by a maximal left ventricular (LV) wall thickness ≥ 15 mm or ≥ 13 mm in individuals with a family history of HCM. Included patients initiated mavacamten between October 2024 and April 2025, excluding those with prior enrollment in mavacamten clinical trials. Additional inclusion criteria comprised: age ≥ 18 years; NYHA functional Class II or III; left ventricular ejection fraction (LVEF) ≥ 55%; and a peak LVOTG at rest or with provocation ≥ 30 mmHg. The study was approved by the Institutional Review Board of Zhongshan Hospital, Fudan University (Approval No. B2025‐304). The investigation conforms to the principles outlined in the Declaration of Helsinki.

### 2.2. Data Collection and Follow‐Up

Baseline patient characteristics, echocardiographic parameters, laboratory data, medication regimens, and comorbidities were collected. NYHA functional class was assessed at baseline and after 12 weeks of mavacamten treatment. Echocardiographic assessments were performed at baseline and after 4, 8, and 12 weeks of mavacamten treatment, with subsequent follow‐up every 3 months afterward. All patients were followed up in outpatient clinics.

### 2.3. Medication Regimen

All patients initiated treatment with mavacamten 2.5 mg once daily.

Four weeks postinitiation: Treatment was suspended if the provoked LVOTG was < 20 mmHg. Patients with provoked LVOTG ≥ 20 mmHg and LVEF ≥ 50% maintained 2.5 mg daily.

At 8 weeks: Patients suspending treatment at Week 4 restarted 2.5 mg daily if LVEF was ≥ 50% without safety concerns. Those continuing 2.5 mg maintained therapy if LVEF remained ≥ 50%.

Twelve‐week assessment:

Dose was increased to 5 mg daily for patients with LVEF ≥ 55% and resting/provoked LVOTG ≥ 30 mmHg.

Current dose was maintained for patients with either:

1. LVEF 50%–55% (any LVOT gradient).
2. LVEF > 55% with resting/provoked LVOTG < 30 mmHg.

Treatment was discontinued permanently if:

1. LVEF was < 50% at any assessment timepoint.
2. Two treatment discontinuations due to LVEF < 50%.

Patients discontinuing due to LVEF < 50% could rechallenge with 2.5 mg after 4 weeks if LVEF recovered to ≥ 50%.

### 2.4. Study Outcomes

The primary outcomes were changes from baseline to final assessment in resting and provoked LVOTG, N‐terminal pro‐B‐type natriuretic peptide (NT‐proBNP) levels, and NYHA functional class.

Safety outcomes encompassed adverse events including LVEF reduction to < 50%, cardiac hospitalization, and all‐cause mortality during follow‐up. Additionally, other complications or side effects that occurred during the entire follow‐up period are considered. Cardiac hospitalization is defined as unplanned hospitalization due to cardiac interventions (pacemaker implantation, septal ethanol ablation, or septal myectomy), arrhythmias (atrial fibrillation, ventricular tachycardia, or ventricular fibrillation), worsening heart failure, or cardiogenic shock.

### 2.5. Statistics

The sample size was estimated to provide 96% power to detect a 50% difference between treatment groups for the primary endpoint, at a two‐sided *p* < 0.05. Statistical analysis was conducted using SPSS Version 30.0. Continuous variables were described as mean ± standard deviation for normally distributed variables or median with interquartile range (IQR) for non‐normally distributed variables. Continuous variables were compared between treatment groups by one‐way or two‐way ANOVA. Categorical measures were reported as frequencies and percentages (%). Categorical variables were analyzed using Fisher′s exact test, whereas continuous variables were evaluated using paired *t*‐tests or nonparametric Mann–Whitney *U* tests to assess changes in outcome variables between baseline and 3‐month follow‐up, as appropriate. *p* value <0.05 was considered significant.

## 3. Results

### 3.1. Study Population

Between October 2024 and April 2025, a total of 72 patients with oHCM were initiated on mavacamten. The baseline characteristics of the study population are presented in Table 1. The mean age of the patients was 60.4 ± 12.5 years, including 47 (65.3%) males. All patients were symptomatic (NYHA class II: *n* = 52, 72.2%; class III: *n* = 20, 27.8%). Medical history included familial HCM (*n* = 5, 6.9%), prior septal myectomy/alcohol septal ablation (*n* = 5, 6.9%), and implanted cardioverter‐defibrillator (*n* = 1, 1.4%). Baseline medications included beta‐blockers (*n* = 46, 63.9%) and nondihydropyridine calcium channel blockers (*n* = 13, 18.1%). Comorbidities comprised atrial fibrillation (*n* = 17, 23.6%) and hypertension (*n* = 26, 36.1%).

**Table 1:** Patient baseline characteristics.

	**Values (** **n** = 72**)**
Age(years)	60.4 ± 12.5
Male	47 (65.3)
Body mass index (kg/m^2^)	24.4 ± 2.5
Systolic blood pressure (mmHg)	126.5 ± 9.9
Diastolic blood pressure (mmHg)	75.5 ± 7.7
Heart rate (bpm)	67.6 ± 5.6
NYHA class	
II	52 (72.2)
III	20 (27.8)
Family history of HCM	5 (6.9)
ICD at baseline	1 (1.4)
Previous septal myectomy/alcohol septal ablation	5 (6.9)
Comorbidities	
Hypertension	26 (36.1)
Diabetes mellitus	4 (5.6)
Atrial fibrillation	17 (23.6)
Coronary artery disease	5 (6.9)
End‐stage renal disease	0(0)
Medications	
Beta‐blocker	46 (63.9)
Calcium channel blocker	13 (18.1)
Oral anticoagulant	12 (16.7)
ACEI/ARB/ARNI	18 (25.0)

*Note:* Values are presented as number (%) or mean ± standard deviation.

Abbreviations: ACEI, angiotensin‐converting enzyme inhibitor; ARB: angiotensin receptor blocker; ARNI, angiotensin receptor‐neprilysin inhibitor; HCM, hypertrophic cardiomyopathy; ICD, implantable cardioverter‐defibrillators; NYHA: New York Heart Association.

### 3.2. Efficacy End Points

At baseline, the average resting LVOTG was 52.4 ± 28.7 mmHg, and the provoked LVOTG was 74.1 ± 24.4 mmHg. Significant reductions in both gradients emerged after 4 weeks of mavacamten therapy and persisted throughout follow‐up. Resting LVOTG decreased to 38.7 ± 27.4 mmHg at 4 weeks, 36.4 ± 25.9 mmHg at 8 weeks, and 32.1 ± 23.1 mmHg at 12 weeks (all *p* < 0.001 vs. baseline), whereas provoked LVOTG declined to 57.0 ± 27.3 mmHg, 52.8 ± 26.0 mmHg, and 48.7 ± 25.4 mmHg at corresponding intervals (all *p* < 0.001). LVEF remained stable, transitioning nonsignificantly from 65.2*%* ± 4.4*%* at baseline to 64.7*%* ± 3.6*%* at 4 weeks, 64.1*%* ± 4.3*%* at 8 weeks, and 64.7*%* ± 4.4*%* at 12 weeks (*p* > 0.05 for all timepoints) (Figure 1). Moreover, the average level of NT‐proBNP decreased from baseline 1102.7 ± 1114.9 pg/mL to 320.2 ± 406.2 pg/mL (*p* < 0.001) at 12 weeks (Figure 1). Beyond LVOTG improvements, 83.3% of patients (60 of 72) experienced ≥ 1‐class improvement in NYHA class at 12 weeks of mavacamten therapy, whereas 12 (16.7%) patients maintained their baseline functional status without deterioration (Figure 1).

Figure 1(a,b) Changes in resting and Valsalva LVOT gradients during follow‐up; (c) changes in LVEF during follow‐up; (d) changes in NYHA class during follow‐up; (e) changes in the level of NT‐proBNP during follow‐up.

**Figure:**
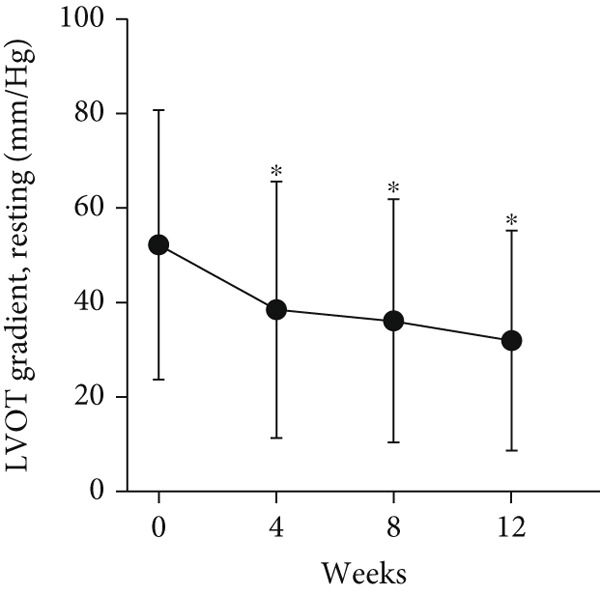
(a)

**Figure:**
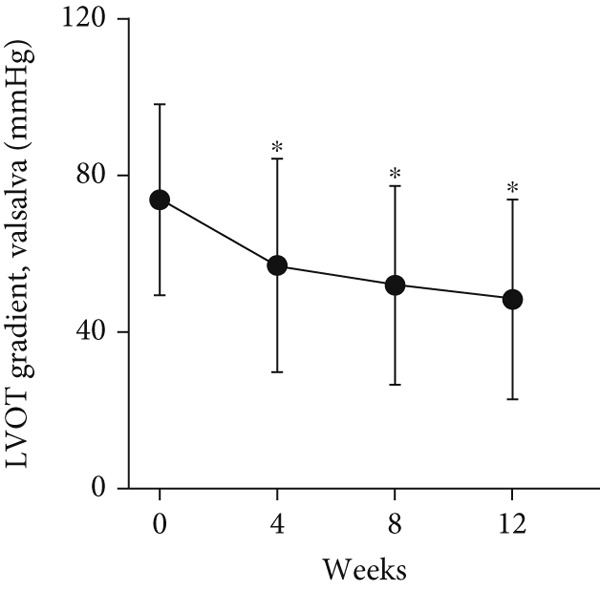
(b)

**Figure:**
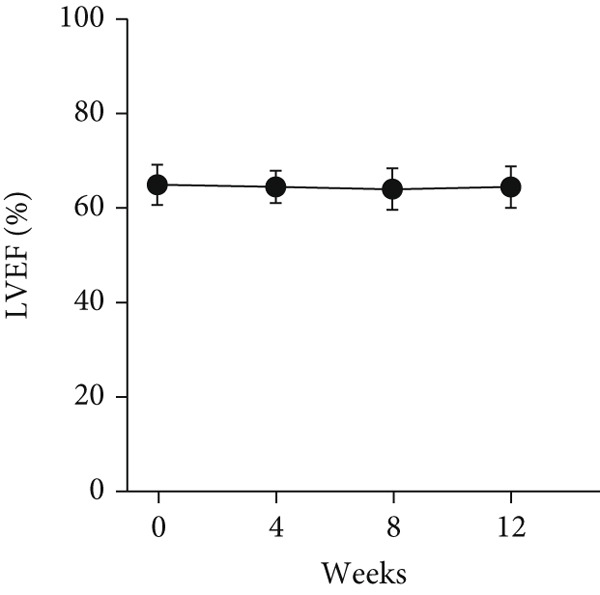
(c)

**Figure:**
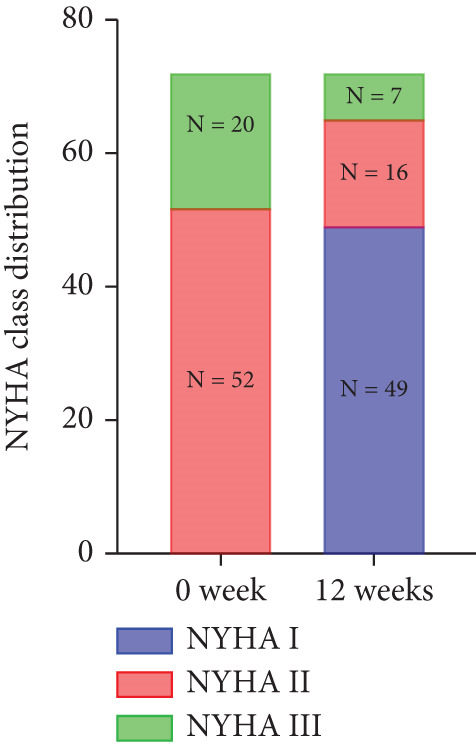
(d)

**Figure:**
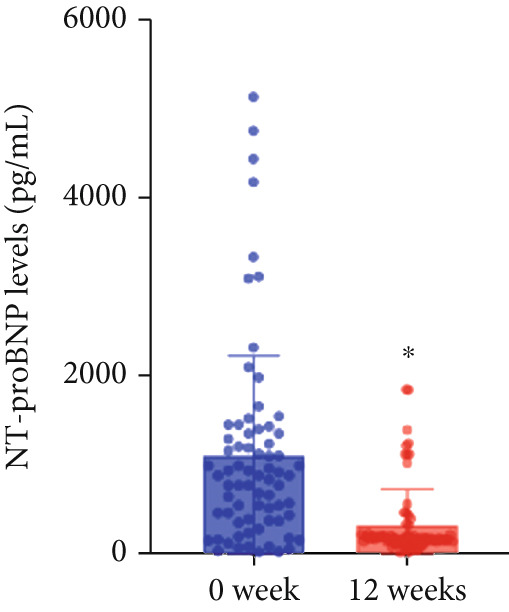
(e)

### 3.3. Subgroup Analysis: Classic HCM (cHCM) and Apical HCM (ApHCM)

The study stratified the mavacamten‐treated cohort into cHCM and ApHCM subgroups, with the latter comprising 39 (54.2%) patients. No intergroup differences existed in baseline resting (ApHCM: 52.3 ± 27.6 mmHg vs. cHCM: 52.5 ± 30.5 mmHg; *p* > 0.05) or provoked (72.1 ± 24.4 mmHg vs. 76.5 ± 24.7 mmHg; *p* > 0.05) LVOTG (Figure 2). At 12 weeks, resting LVOTG decreased significantly from baseline in both ApHCM (52.3 ± 27.6 to 40.2 ± 24.7 mmHg, *p* < 0.001) and cHCM (52.5 ± 30.5 to 22.4 ± 16.9 mmHg, *p* < 0.001) subgroups. Similarly, provoked gradients declined from 72.1 ± 24.4 to 58.7 ± 26.1 mmHg in ApHCM patients and from 76.5 ± 24.7 to 36.8 ± 18.7 mmHg in cHCM patients (all *p* < 0.001) (Figure 2). The mean reduction in resting LVOTG from baseline to week 12 was significantly greater in cHCM patients (30.1 ± 25.9 mmHg) compared with ApHCM patients (12.1 ± 17.1 mmHg; *p* < 0.001). Similarly, the decrease in provoked LVOTG was more pronounced in the cHCM group (39.7 ± 22.6 mmHg) versus the ApHCM group (13.4 ± 17.4 mmHg; *p* < 0.001) (Figure 2). Both subgroups demonstrated no significant reduction in LVEF from baseline to 12 weeks of mavacamten therapy (*p* > 0.05) (Figure 2). No intergroup differences existed in baseline level of NT‐proBNP (ApHCM: 1150.6 ± 1032.0 pg/mL vs. cHCM: 1046.0 ± 1219.5 pg/mL; *p* > 0.05). At 12 weeks, the average level of NT‐proBNP decreased significantly from baseline in both ApHCM (1150.6 ± 1032.0 to 337.0 ± 375.6 pg/mL, *p* < 0.001) and cHCM (1046.0 ± 1219.5 to 300.5 ± 444.7 pg/mL, *p* < 0.001) subgroups. The mean reduction in NT‐proBNP from baseline to week 12 was similar in cHCM patients (813.6 ± 742.4 pg/mL) compared with ApHCM patients (745.5 ± 830.0 pg/mL; *p* < 0.001) (Figure 2). In the cHCM group, 84.8% of patients (28 of 33) showed ≥ 1 NYHA class improvement at 12 weeks, whereas in the ApHCM group it was 82.1% (32 of 39), with no statistically significant difference between the two groups (Figure 2).

Figure 2(a,b) Changes in resting and Valsalva LVOT gradients during follow‐up; (c) changes in LVEF during follow‐up; (d,e) changes in the mean reduction in resting and Valsalva LVOT gradients during follow‐up; (f) changes in the level of NT‐proBNP during follow‐up; (g) changes in NYHA class during follow‐up.

**Figure:**
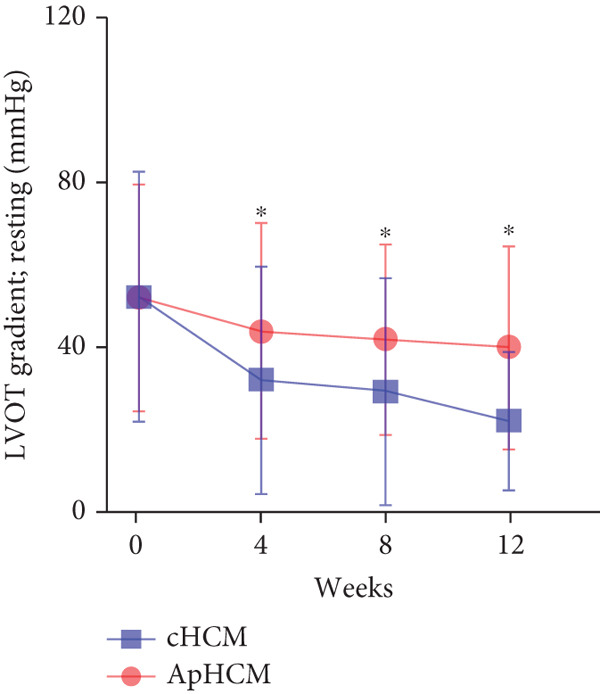
(a)

**Figure:**
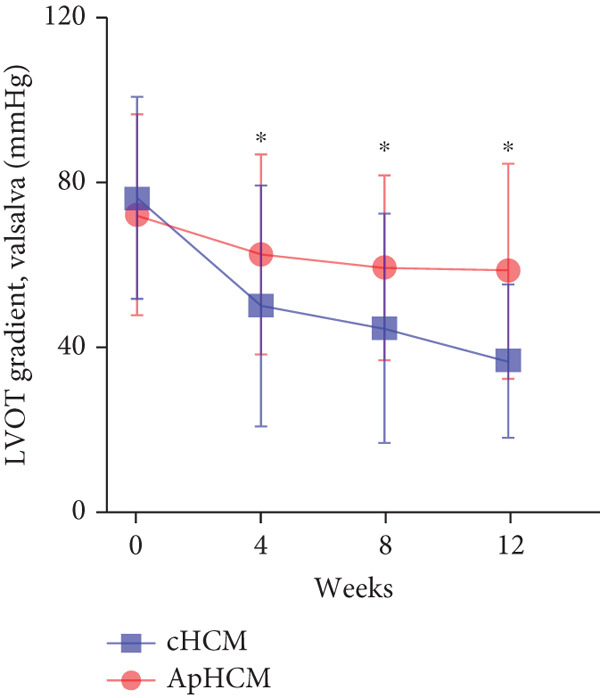
(b)

**Figure:**
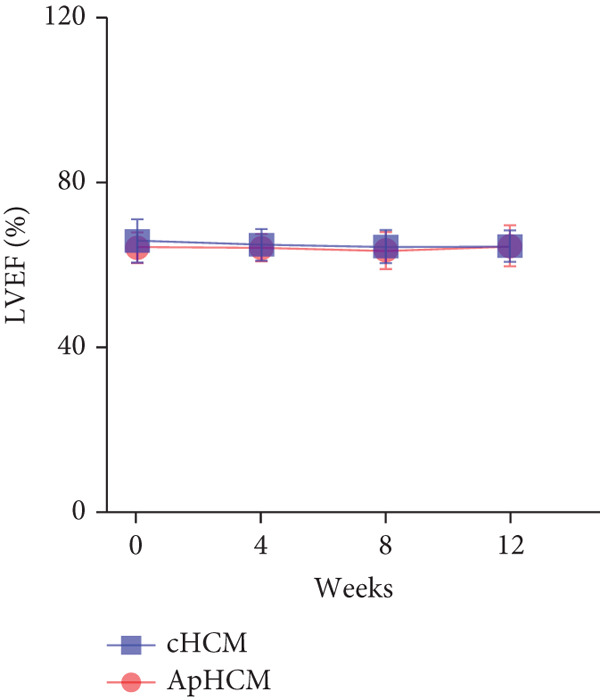
(c)

**Figure:**
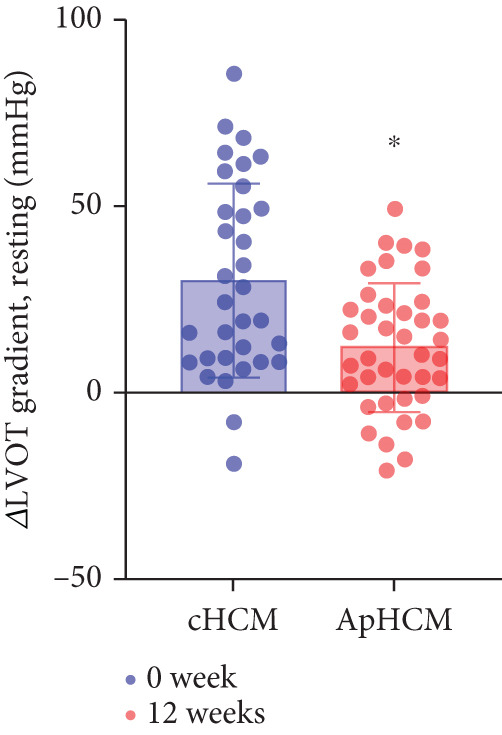
(d)

**Figure:**
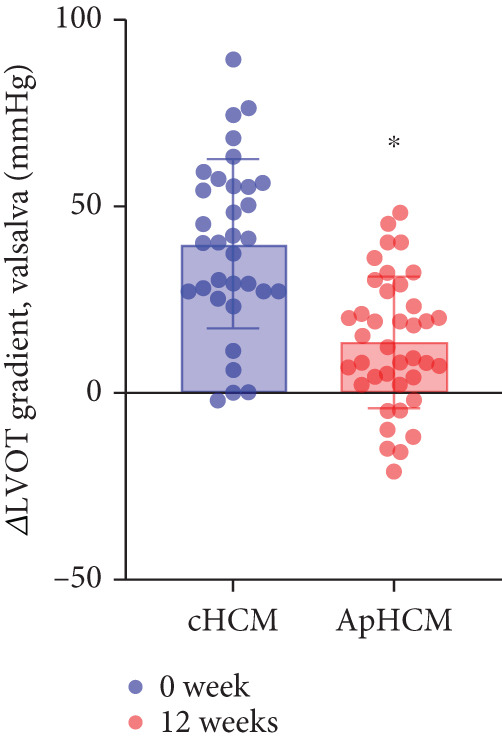
(e)

**Figure:**
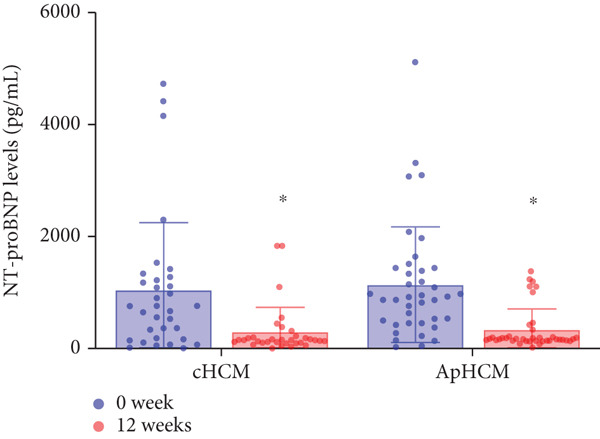
(f)

**Figure:**
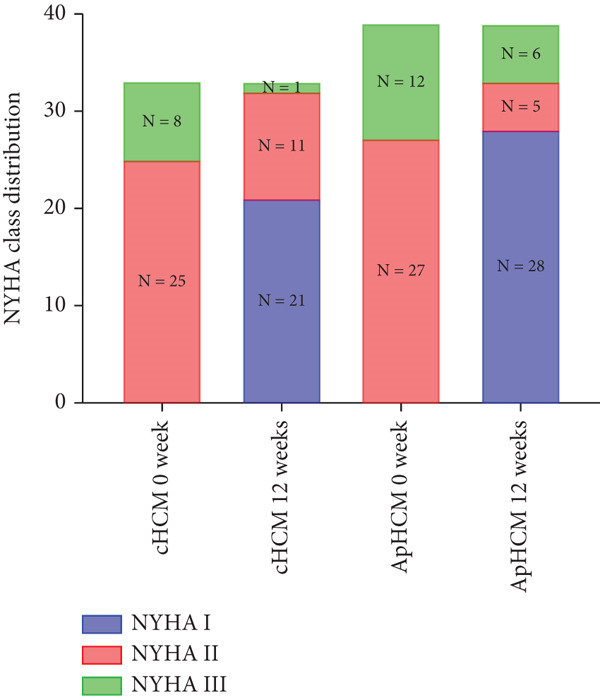
(g)

### 3.4. Subgroup Analysis: Low LVOTG (L‐LVOTG) Group and High LVOTG (H‐LVOTG) Group

Patients receiving mavacamten were further stratified according to resting LVOTG into two subgroups: L‐LVOTG group (< 50 mmHg; *n* = 38) and H‐LVOTG group (≥ 50 mmHg; *n* = 34). At 12 weeks, resting LVOTG decreased significantly from baseline in both subgroups: L‐LVOTG (29.7 ± 11.5 to 22.0 ± 14.8 mmHg; *p* < 0.001) and H‐LVOTG (77.8 ± 19.2 to 43.4 ± 25.7 mmHg; *p* < 0.001). Similarly, provoked gradients declined from 58.3 ± 18.5 to 38.2 ± 18.4 mmHg in L‐LVOTG patients and from 91.9 ± 17.0 to 60.4 ± 27.1 mmHg in H‐LVOTG patients (both *p* < 0.001; Figure 3). The mean reduction in resting LVOTG was significantly greater in H‐LVOTG patients (34.5 ± 24.2 mmHg) versus L‐LVOTG patients (7.7 ± 13.0 mmHg; *p* < 0.001). Similarly, provoked gradient reductions were larger in the H‐LVOTG group (31.4 ± 23.8 mmHg) compared with the L‐LVOTG group (20.1 ± 22.8 mmHg; *p* = 0.04; Figure 3). Both subgroups demonstrated no significant reduction in LVEF from baseline to 12 weeks of mavacamten therapy (*p* > 0.05) (Figure 3). NT‐proBNP levels decreased significantly in both subgroups at 12 weeks (L‐LVOTG: 993.7 ± 1013.9 to 303.4 ± 402.8 pg/mL; H‐LVOTG: 1224.5 ± 1221.8 to 339.1 ± 415.2 pg/mL; both *p* < 0.001). NYHA class improvement (≥ 1 class) was observed in 84.2% (32/38) of L‐LVOTG patients and 82.4% (28/34) of H‐LVOTG patients (*p* = NS; Figure 3).

Figure 3(a,b) Changes in resting and Valsalva LVOT gradients during follow‐up; (c) changes in LVEF during follow‐up; (d,e) changes in the mean reduction in resting and Valsalva LVOT gradients during follow‐up; (F) changes in the level of NT‐proBNP during follow‐up; (g) changes in NYHA class during follow‐up.

**Figure:**
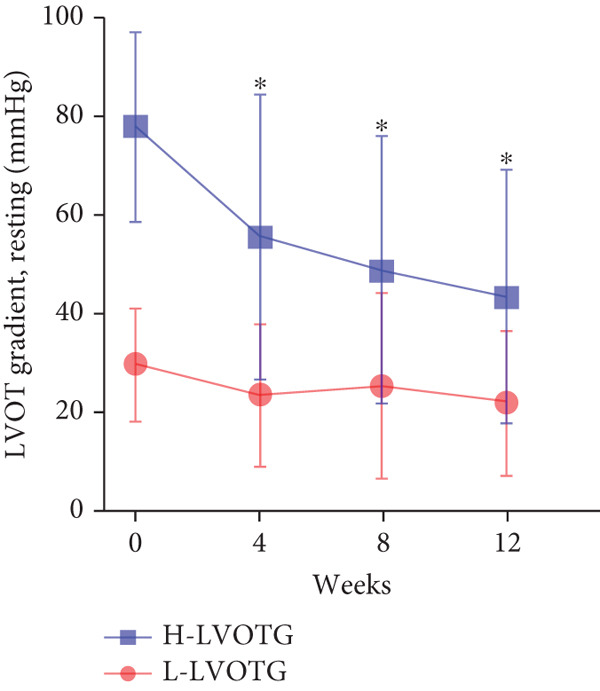
(a)

**Figure:**
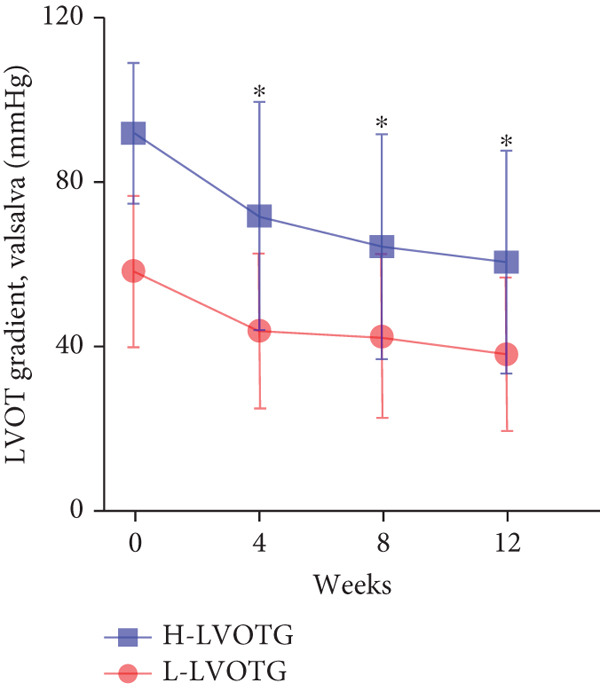
(b)

**Figure:**
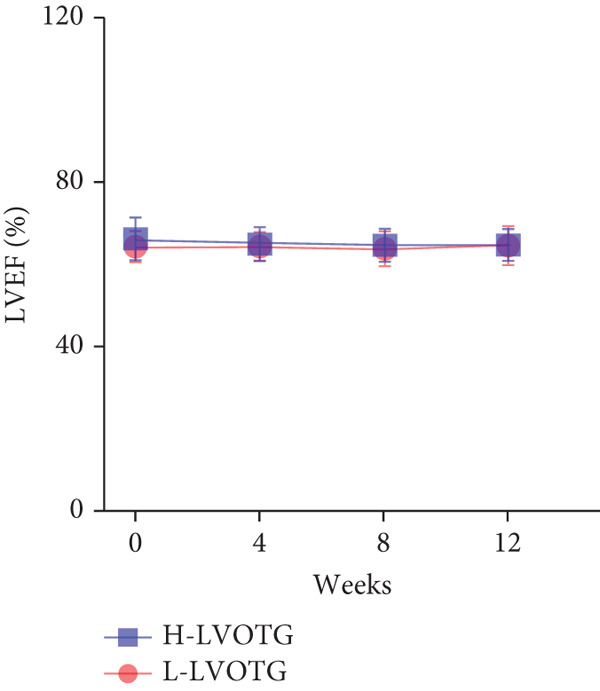
(c)

**Figure:**
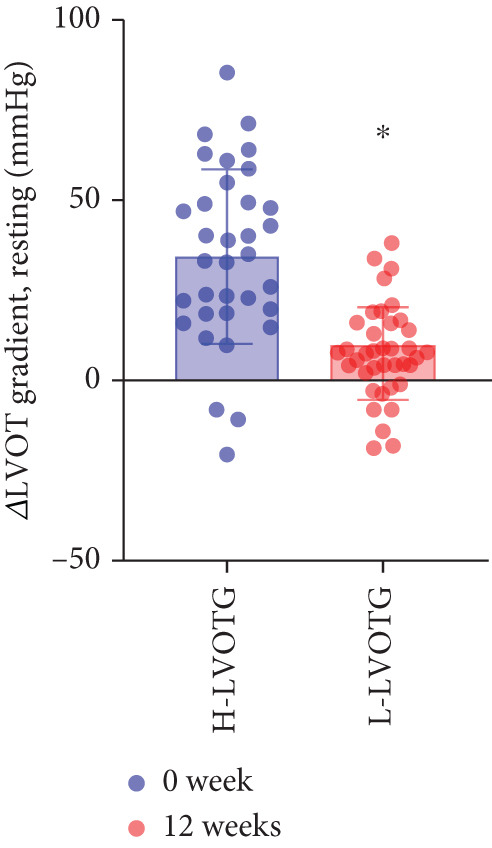
(d)

**Figure:**
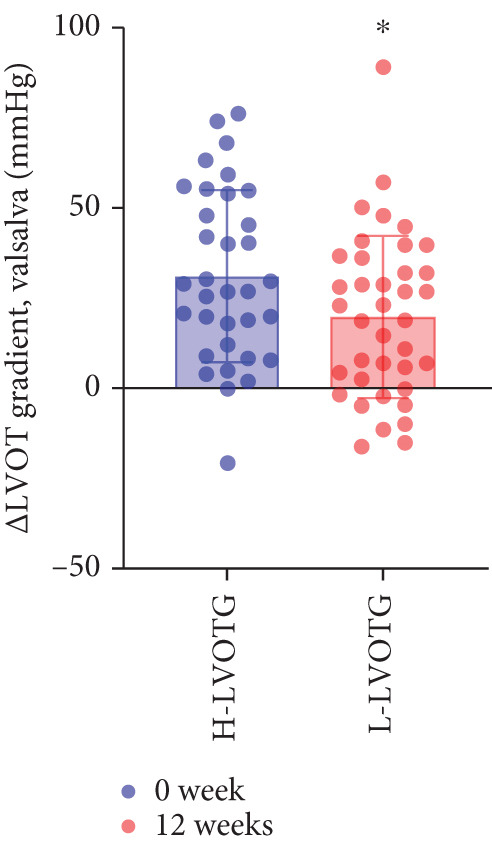
(e)

**Figure:**
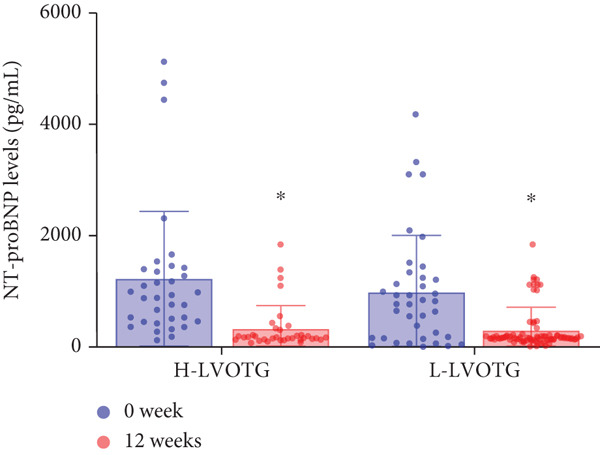
(f)

**Figure:**
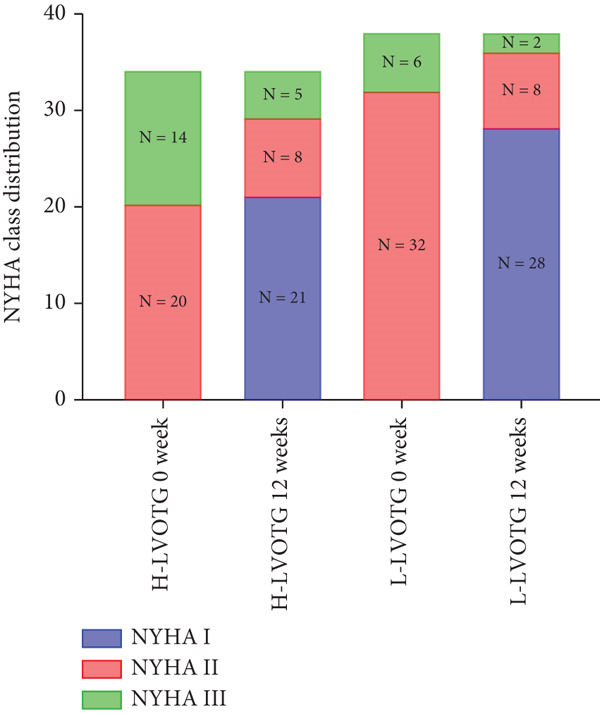
(g)

### 3.5. Subgroup Analysis: LVOTO and Mid‐Left Ventricular Obstruction (Mid‐LVO)

We further stratified mavacamten‐treated oHCM patients into two obstruction phenotype subgroups: LVOTO (*n* = 61) and mid‐LVO (*n* = 11) for comparative analysis. No baseline differences existed in resting (LVOTO: 52.8 ± 29.7 mmHg vs. mid‐LVO: 50.5 ± 23.7 mmHg; *p* > 0.05) or provoked (74.4 ± 25.7 mmHg vs. 72.6 ± 17.1 mmHg; *p* > 0.05) gradients (Figure 4). At 12 weeks, resting gradients decreased significantly in LVOTO patients (52.8 ± 29.7 to 30.8 ± 23.9 mmHg; *p* < 0.001) but not in mid‐LVO patients (50.5 ± 23.7 to 39.2 ± 17.8 mmHg; *p* = 0.07). Similarly, provoked gradients declined significantly in LVOTO patients (74.4 ± 25.7 to 47.1 ± 26.6 mmHg; *p* = 0.01) versus nonsignificantly in mid‐LVO patients (72.6 ± 17.1 to 57.6 ± 15.1 mmHg; *p* = 0.18) (Figure 4). The mean reduction in resting gradients was no statistically significant difference in LVOTO versus mid‐LVO (22.0 ± 23.6 vs. 11.3 ± 20.0 mmHg; *p* = 0.13), with a similar trend for provoked gradients (27.3 ± 24.5 vs. 15.1 ± 17.2 mmHg; *p* = 0.06) (Figure 4). Both subgroups demonstrated no significant reduction in LVEF from baseline to 12 weeks of mavacamten therapy (*p* > 0.05) (Figure 4). At 12 weeks, NT‐proBNP levels decreased significantly in both subgroups at 12 weeks (LVOTO: 1089.7 ± 1070.4 to 302.7 ± 374.9 pg/mL; mid‐LVO: 1174.5 ± 1394.4 to 323.4 ± 414.4 pg/mL; both *p* < 0.001). NYHA class improvement (≥ 1 class) occurred in 83.6% (51/61) of LVOTO and 81.8% (9/11) of mid‐LVO patients (*p* = NS) (Figure 4).

Figure 4(a,b) Changes in resting and Valsalva LVOT gradients during follow‐up; (c) changes in LVEF during follow‐up; (d,e) changes in the mean reduction in resting and Valsalva LVOT gradients during follow‐up; (f) changes in the level of NT‐proBNP during follow‐up; (g) changes in NYHA class during follow‐up.

**Figure:**
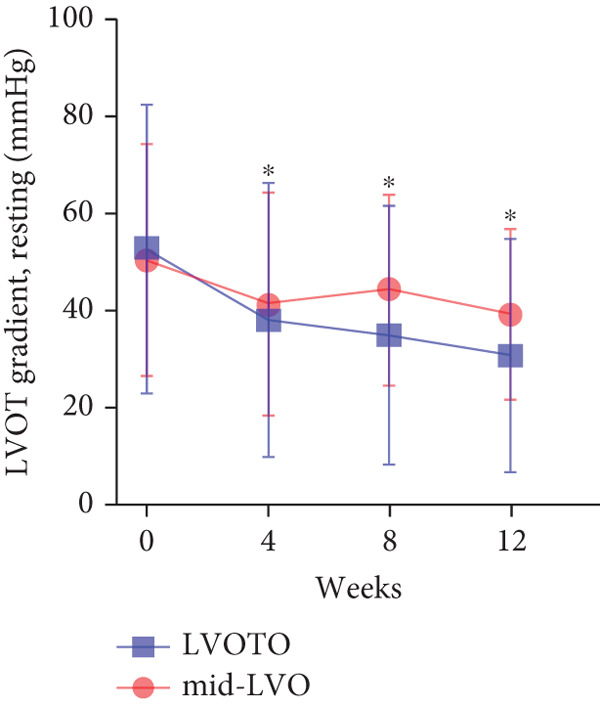
(a)

**Figure:**
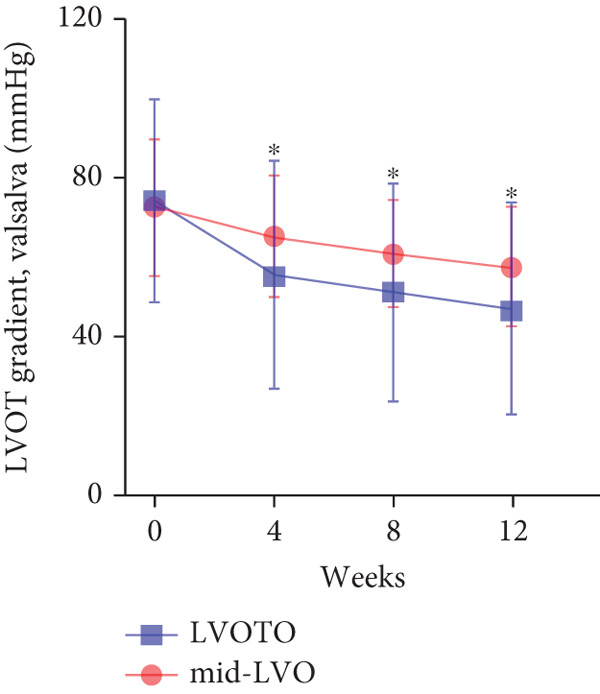
(b)

**Figure:**
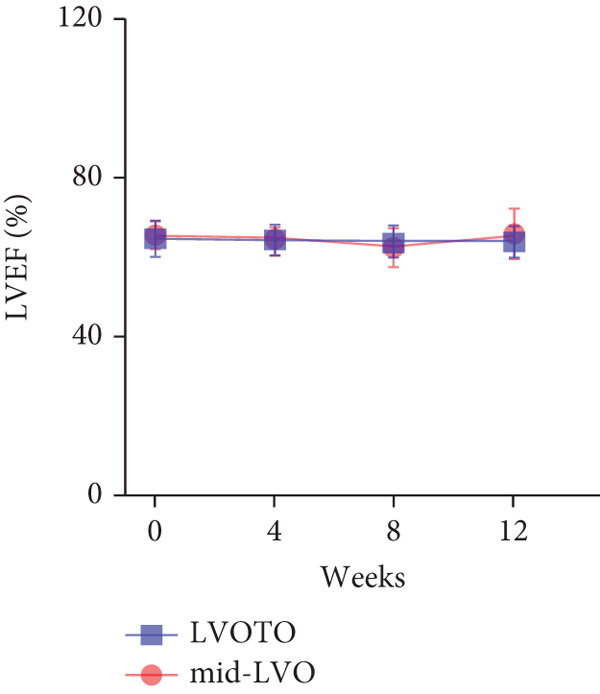
(c)

**Figure:**
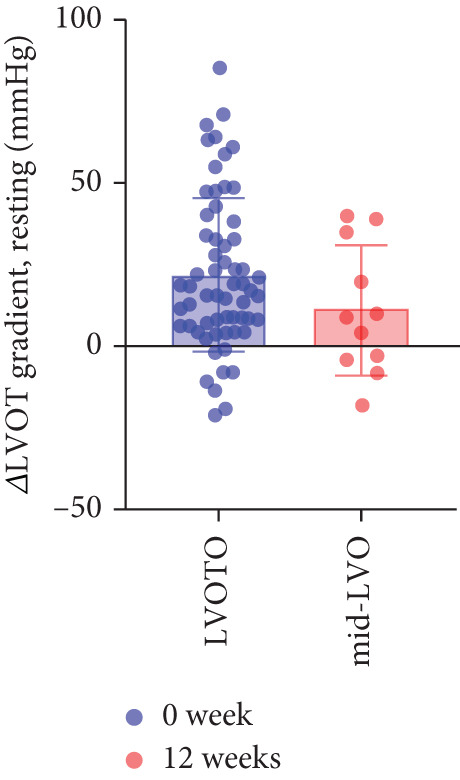
(d)

**Figure:**
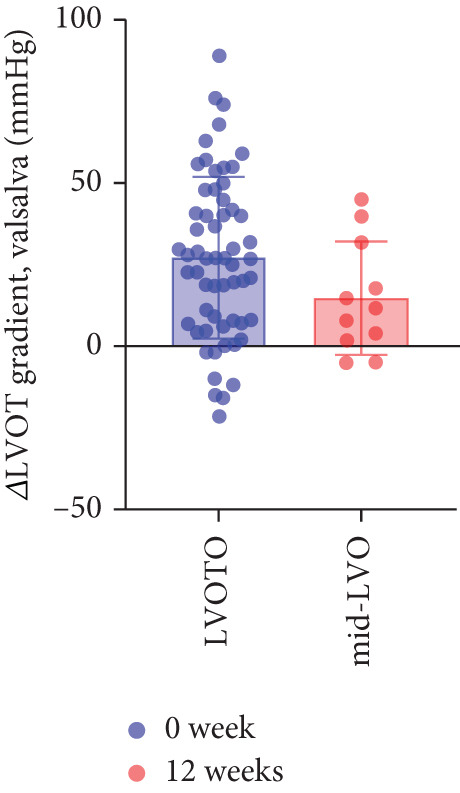
(e)

**Figure:**
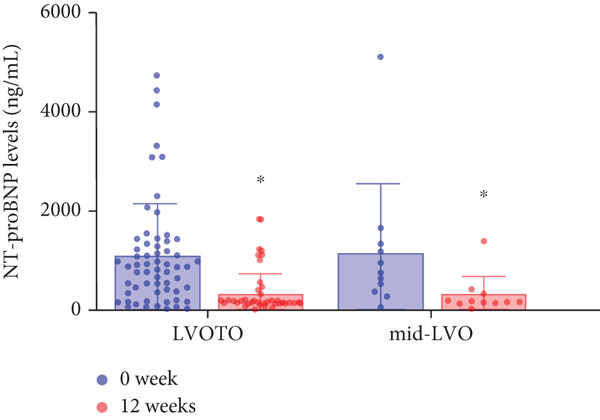
(f)

**Figure:**
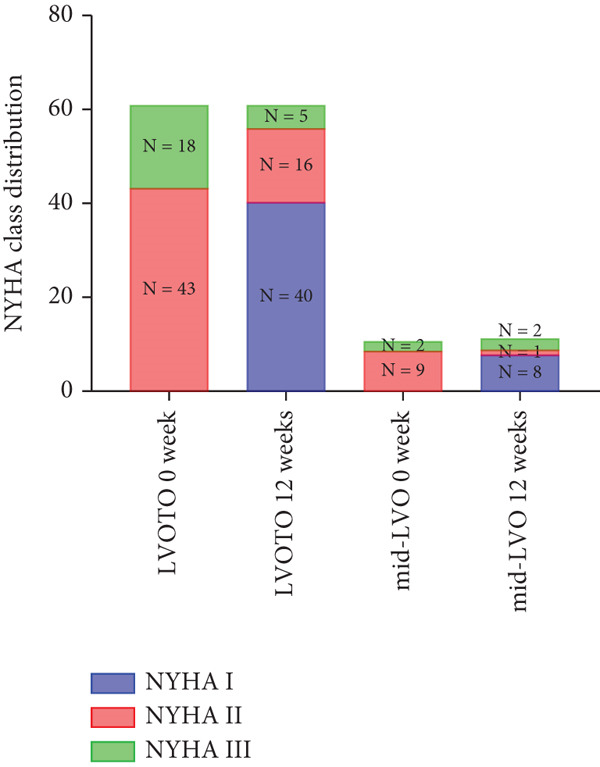
(g)

### 3.6. Doses Adjustment

After 12 weeks of treatment, dose was increased to 5 mg for patients with LVEF ≥ 55% and resting/provoked LVOTG ≥ 30 mmHg. Due to financial constraints and insurance limitations, 22 patients received an increased dosage to 5 mg of mavacamten. This cohort comprised eight cHCM and 14 ApHCM patients, with 11 patients each in the L‐LVOTG and H‐LVOTG subgroups. The mid‐LVOTG subgroup (*n* = 3) was excluded from comparative analyses due to insufficient sample size following dose adjustment. Increasing the Mava dosage to 5 mg resulted in further significant reductions in both resting and provoked LVOTG in patients. Specifically, the resting LVOTG decreased significantly from 38.6 ± 23.8 to 23.5 ± 15.9 mmHg (*p* < 0.001), whereas the provoked LVOTG decreased significantly from 59.0 ± 27.3 to 39.6 ± 19.4 mmHg (*p* < 0.001) (Figure 5). Subgroup analysis revealed that in the cHCM subgroup, the resting LVOTG decreased from 17.8 ± 11.6 to 12.8 ± 5.0 mmHg (*p* = 0.10), and the provoked LVOTG decreased from 37.9 ± 16.4 to 31.1 ± 14.7 mmHg (*p* = 0.08), and in the ApHCM subgroup, the resting LVOTG decreased significantly from 50.5 ± 20.5 to 29.6 ± 16.8 mmHg (*p* < 0.01), and the provoked LVOTG decreased significantly from 71.1 ± 25.1 to 44.5 ± 20.6 mmHg (*p* < 0.01)(Figure 5). Analysis by baseline gradient severity revealed that in the L‐LVOTG subgroup, the resting LVOTG decreased significantly from 29.3 ± 16.7 to 19.3 ± 13.4 mmHg (*p* = 0.02), whereas the provoked LVOTG decreased from 51.3 ± 16.9 to 38.7 ± 18.3 mmHg (*p* = 0.07). In the H‐LVOTG subgroup, both the resting LVOTG (47.9 ± 26.8 to 27.6 ± 17.7 mmHg; *p* < 0.01) and provoked LVOTG (66.7 ± 34.0 to 42.6 ± 22.7 mmHg; *p* < 0.01) decreased significantly (Figure 5). Notably, following the increased Mava dosage to 5 mg, the patient′s LVEF did not exhibit a significant decrease (65.1 ± 5.1*%* vs. 63.6 ± 3.6*%*; *p* > 0.05)(Figure 5).

Figure 5(a–k) Changes in resting and Valsalva LVOT gradients during follow‐up.

**Figure:**
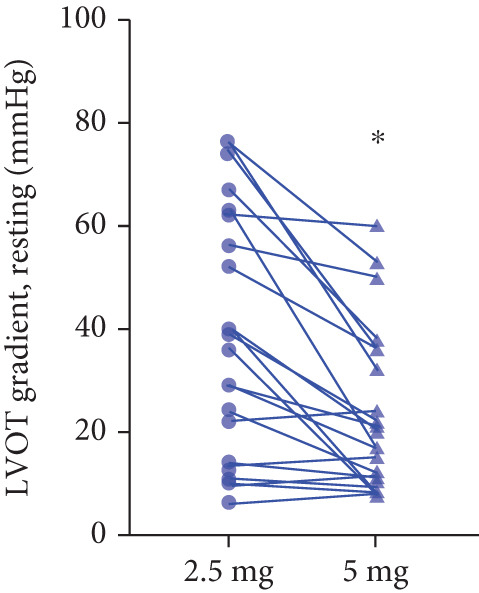
(a)

**Figure:**
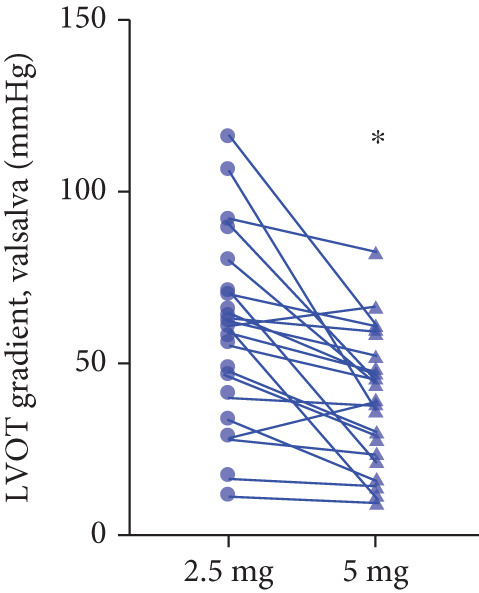
(b)

**Figure:**
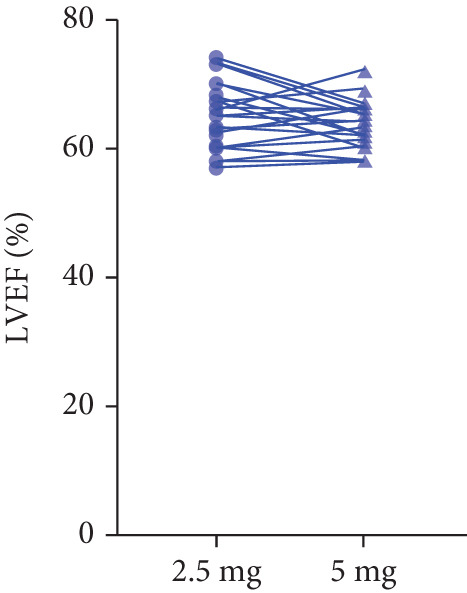
(c)

**Figure:**
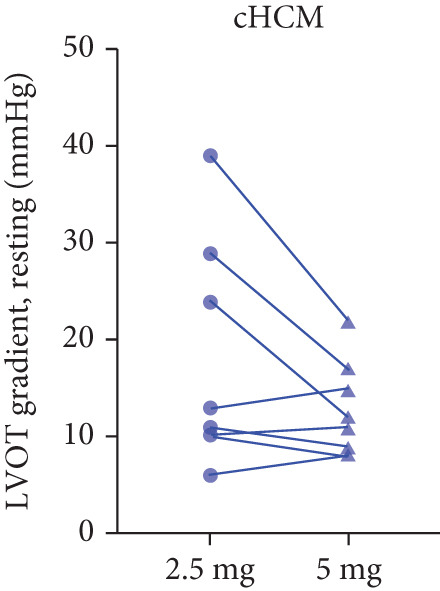
(d)

**Figure:**
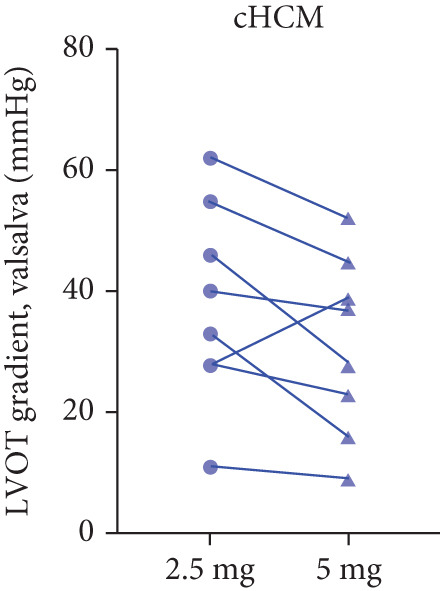
(e)

**Figure:**
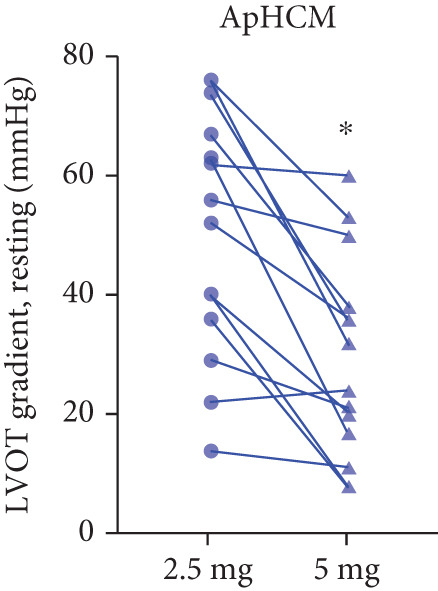
(f)

**Figure:**
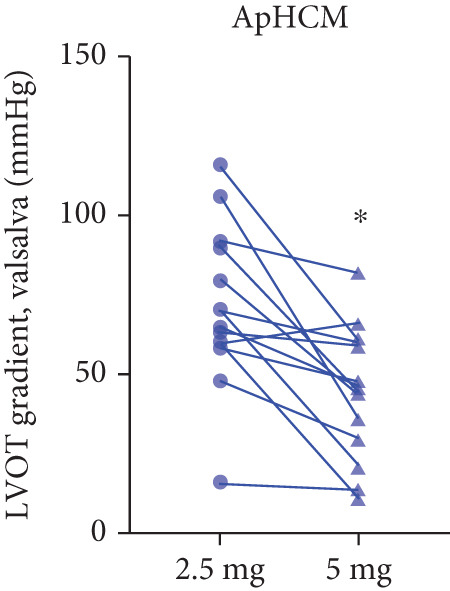
(g)

**Figure:**
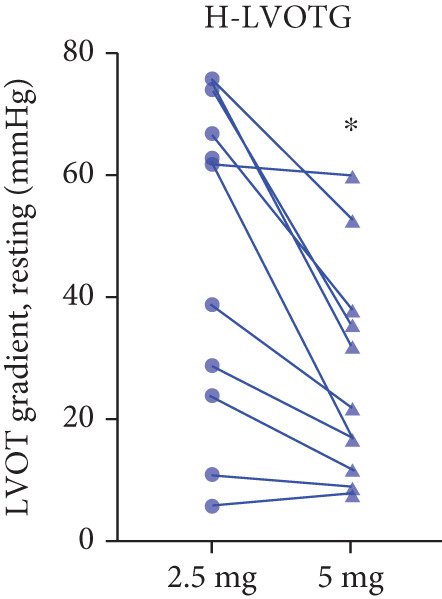
(h)

**Figure:**
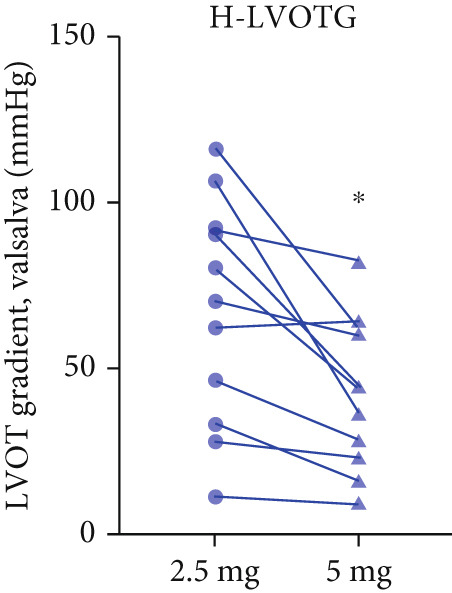
(i)

**Figure:**
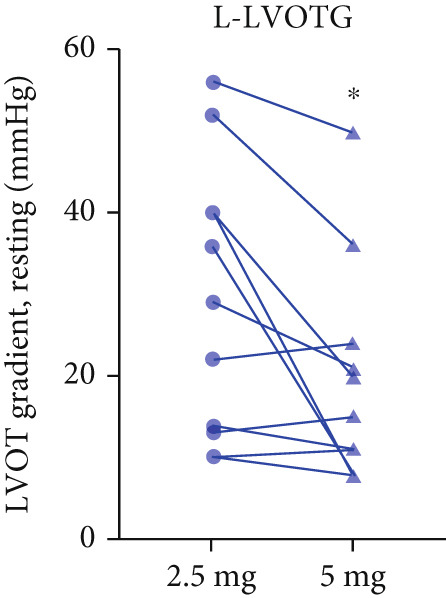
(j)

**Figure:**
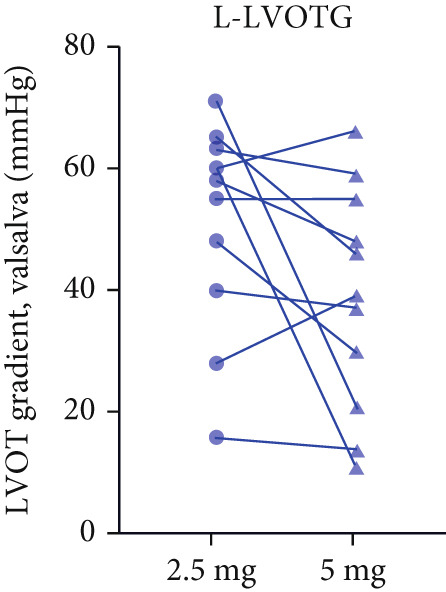
(k)

### 3.7. Safety

Throughout the treatment period, when starting with a dose of 2.5 mg, no reduction in LVEF < 50% was observed in any patient following initiation of therapy. Furthermore, no new‐onset arrhythmias, cardiac hospitalizations, or deaths occurred in the study population. Similarly, among the 22 patients whose dose was increased to 5 mg, no instances of these adverse outcomes were observed. Mava was generally well tolerated. Minor adverse events, including dizziness, nausea, and fatigue, were reported in two patient receiving 2.5 mg and two patients receiving 5 mg. All symptoms resolved spontaneously within several weeks without requiring treatment discontinuation.

## 4. Discussion

This real‐world observational study provides the first clinical evidence of mavacamten′s efficacy and safety in Chinese patients with symptomatic oHCM, addressing a critical gap in data from Asian populations. Our findings demonstrate that mavacamten, initiated at 2.5 mg daily, significantly improves the resting and Valsalva LVOT gradients, NT‐proBNP levels, and subjective symptoms within 12 weeks while maintaining a favorable safety profile.

This study demonstrated comparable age and gender distributions to the EXPLORER‐HCM and VALOR‐HCM trials but lower BMI, consistent with the known BMI profile of Asian populations as evidenced by similarity to the EXPLORER‐CN study [9–11, 13]. Unlike EXPLORER‐HCM, we exclusively utilized the Valsalva maneuver to provoke LVOT gradients. Prior studies have demonstrated a strong correlation between Valsalva‐induced and exercise‐induced LVOT gradients in HCM patients with resting obstruction. This approach adheres to Chinese guidelines, which recommend Valsalva as an alternative provocation method [14, 15]. Notably, despite the higher prevalence of CYP2C19 poor metabolizers in Asians, EXPLORER‐CN demonstrated consistent LVOT gradient reduction across all CYP2C19 phenotypes [16, 17]. Thus, we followed a pharmacokinetic and pharmacodynamic‐guided titration protocol without CYP2C19 genotyping, initiating mavacamten at 2.5 mg.

Baseline resting and Valsalva LVOT gradients in our cohort (52.4 ± 28.7 and 74.1 ± 24.4 mmHg, respectively) were similar to those in EXPLORER‐HCM (resting: 52 ± 29 mmHg; Valsalva: 72 ± 32 mmHg) and VALOR‐HCM (resting: 51.2 ± 31.4 mmHg; Valsalva: 75.3 ± 30.8 mmHg). However, they were lower than in EXPLORER‐CN (resting: 74.6 ± 35.1 mmHg; Valsalva: 106.8 ± 43.2 mmHg) and a Korean real‐world study (resting: 58.4 ± 46.4 mmHg; Valsalva: 92.6 ± 46.8 mmHg) [18]. Mavacamten treatment significantly reduced both resting and provoked LVOT gradients from baseline within 4 weeks, with progressive reductions sustained through Week 12. At Week 12, mean reductions were 20.4 mmHg (resting) and 25.4 mmHg (provoked), indicating early statistically significant hemodynamic benefits. In contrast, EXPLORER‐HCM reported larger reductions at Week 12 (resting: 35.6 mmHg; provoked: 44.0 mmHg), whereas EXPLORER‐CN showed reductions of 34.9 mmHg (resting) and 38.6 mmHg (provoked) at the same timepoint. VALOR‐HCM (Week 16: resting *Δ*36.0 mmHg; provoked *Δ*45.2 mmHg) and the Korean real‐world study (resting *Δ*40.1 mmHg; provoked *Δ*68.1 mmHg) lacked Week 12 data. The comparatively smaller reductions in resting and provoked LVOT gradients at Week 12 in this study relative to EXPLORER‐HCM may be attributable to lower mavacamten dosing. Dose escalation to 5 mg after Week 12 yielded further hemodynamic improvement, with additional mean reductions of 15.1 mmHg in resting gradient and 19.4 mmHg in provoked gradient, demonstrating that while low‐dose mavacamten (2.5 mg) achieves significant gradient reduction, dose optimization enhances therapeutic efficacy. The observed differential efficacy versus VALOR‐HCM and Korean real‐world data may reflect both lower initial dosing and shorter treatment duration. This interpretation is supported by EXPLORER‐CN data showing progressive benefit with extended therapy: at 30 weeks (starting dose 2.5 mg), gradient reductions reached 49.0 mmHg (resting) and 70.3 mmHg (provoked), exceeding outcomes in shorter‐duration studies. Notably, Asian populations exhibit both lower average BMI and potentially enhanced hemodynamic response to mavacamten, as evidenced by greater gradient reductions achieved with lower doses in this demographic compared with Western cohorts, suggesting possible ethnic variations in drug sensitivity [9, 18, 19].

The comparatively smaller reductions in resting and provoked LVOT gradients at Week 12 versus the EXPLORER‐CN study are likely attributable to lower baseline gradients in our cohort. Given established hemodynamic thresholds—where gradients ≥ 30 mmHg define obstructive HCM and ≥ 50 mmHg indicate eligibility for septal reduction therapy (SRT) [6, 20]. Patients in H‐LVOTG subgroup demonstrated significantly greater reductions than those in L‐LVOTG subgroup: mean resting gradient decreased by 34.5 mmHg versus 7.7 mmHg, whereas provoked gradients decreased by 31.5 mmHg versus 20.1 mmHg, respectively. These findings demonstrate mavacamten′s enhanced efficacy in patients with more severe baseline obstruction, suggesting gradient magnitude may serve as a key predictor of therapeutic response.

Low‐dose mavacamten significantly reduced NT‐proBNP levels at Week 12 in our cohort consistent with prior studies. This reduction corresponds with improved cardiac remodeling parameters including decreased left ventricular mass index, absolute left ventricular mass, left atrial volume index, and maximal septal wall thickness, suggesting that NT‐proBNP reductions may be mediated through attenuated left ventricular wall stress and myocardial injury [11, 21]. Supporting this mechanistic relationship, Korean real‐world data demonstrate a linear correlation between LVOT gradient reduction and NT‐proBNP changes, indicating NT‐proBNP′s potential utility as a dynamic biomarker for monitoring hemodynamic response [18].

Notably, > 80% of patients exhibited ≥ 1‐class NYHA improvement—significantly higher than reported in other trials. This enhanced clinical response may reflect both lower baseline hemodynamic burden and possible ethnic variations in drug sensitivity, consistent with our earlier observations of greater gradient reductions in Asian populations [11, 18, 19].

ApHCM, a morphological subtype characterized by predominant apical involvement, electrocardiographic giant negative T‐waves, and a “spade‐like” left ventricular cavity on ventriculography, demonstrates higher prevalence in Asian populations (up to 40% of HCM cases) [22]. In this study, mavacamten‐treated patients were stratified into cHCM and ApHCM subgroups. While baseline resting and provoked gradients showed no significant differences, both groups achieved statistically significant reductions at Week 12. However, ApHCM patients exhibited substantially smaller reductions in resting and provoked gradients compared with cHCM patients, indicating diminished hemodynamic response. This differential efficacy may reflect ApHCM′s distinct pathophysiology: lower sarcomeric mutation prevalence, frequent mid‐ventricular hypertrophy causing mid‐cavitary obstruction [23, 24], and more severe microvascular dysfunction and myocardial fibrosis factors potentially limiting mavacamten′s mechanism‐based reduction of outflow tract obstruction [22, 25, 26].

In addition, further subgroup analysis based on different obstructive sites in this study partially illustrates this issue. Further subgroup analysis by obstruction site revealed that while both LVOTO and mid‐LVO groups demonstrated comparable baseline resting and provoked gradients, both achieved statistically significant reductions at Week 12. Notably, the mid‐LVO subgroup exhibited numerically smaller reductions in resting and provoked gradients compared with LVOTO patients, with a clinically relevant difference trend that did not reach statistical significance likely due to MVO′s limited sample size, suggesting potentially diminished mavacamten efficacy in mid‐cavitary obstruction that warrants validation in larger cohorts. Importantly, dose escalation to 5 mg further significantly reduced gradients in ApHCM patients, though mid‐LVO‐dose‐response analysis was precluded by small numbers. These findings indicate that for ApHCM patients with suboptimal response to initial low‐dose therapy, dose titration should be considered prior to concluding drug inefficacy.

Notably, despite attenuated hemodynamic responses in ApHCM and mid‐LVO subgroups, mavacamten comparably improved NYHA class and reduced NT‐proBNP levels across all obstruction sites and HCM subtypes. This consistent clinical benefit, observed irrespective of obstruction sites and HCM subtypes, suggests symptom relief is likely mediated through mavacamten′s dual mechanisms of reducing myocardial contractility and improving cardiac remodeling [7, 21]. Throughout the study period, no patients exhibited LVEF decline below 50%, and there were zero cardiac hospitalizations or deaths, aligning with the safety profile reported in the EXPLORER‐CN trial [11]. These findings collectively demonstrate that low‐dose mavacamten maintains favorable tolerability while providing clinically meaningful functional improvement in Chinese oHCM patients, even in subgroups with diminished gradient reduction.

In conclusion, mavacamten initiation at 2.5 mg significantly improved hemodynamic parameters (resting and provoked LVOT gradients), functional status (NYHA class), and biomarkers (NT‐proBNP) in Chinese patients with oHCM, demonstrating a favorable safety profile with no LVEF reduction below 50% or major cardiac events. Therapeutic efficacy was most pronounced in cHCM and high‐gradient subgroups, whereas dose escalation to 5 mg provided additional hemodynamic benefit. These findings reinforce mavacamten as a transformative therapy for oHCM in Asian populations.

## 5. Limitations

This single‐center observational study is limited by its modest sample size and relatively short follow‐up duration, necessitating validation in larger, multicenter cohorts with extended observation periods. Cost considerations constrained the number of patients receiving dose escalation to ≥ 5 mg of mavacamten. Additionally, genetic analysis was not performed due to limited accessibility and acceptability in China.

## Conflicts of Interest

The authors declare that they have no known competing financial interests or personal relationships that could have appeared to influence the work reported in this paper.

## Author Contributions

Wenlong Yang, Hui Shi, and Rebecca Suchi Chang contributed equally to this work.

## Funding

This study was supported by the National Natural Science Foundation of China (10.13039/501100001809), (82200297, 82200393); Natural Science Foundation of Shanghai (21ZR1413500); Shanghai Top Priority center construction project (2022ZZ01010); Shanghai Clinical Research Center for Interventional Medicine (19MC1910300).

## Data Availability

The data that support the findings of this study are available on request from the corresponding author. The data are not publicly available due to privacy or ethical restrictions.
